# Assessment of dental caries in children with organic lesions of the nervous system using ICDAS II criteria

**DOI:** 10.25122/jml-2020-0155

**Published:** 2021

**Authors:** Khrystyna Vasylivna Pryimak, Iryna Anatoliivna Zoriy, Nataliia Vasylivna Bidenko, Anatoliy Vasylovych Borysenko, Viktor Markiyanovich Batig, Tetiana Anatoliyivna Hlushchenko, Iryna Viktorivna Batih, Michael Ivanovich Sheremet

**Affiliations:** 1. Department of Pediatric and Preventive Dentistry, Bogomolets National Medical University, Kyiv, Ukraine; 2. Department of Nervous Diseases, Psychiatry and Medical Psychology, Bukovinian State Medical University, Chernivtsi, Ukraine; 3. Head of the Department of Therapeutic Dentistry, Bogomolets National Medical University, Kyiv, Ukraine; 4. Department of Therapeutic Dentistry, Bukovinian State Medical University, Chernivtsi, Ukraine; 5. Department of Pediatric Dentistry, Bukovinian State Medical University, Chernivtsi, Ukraine; 6. Surgery Department No. 1, Bukovinian State Medical University, Chernivtsi, Ukraine

**Keywords:** KEYWORDS, cerebral palsy, dental caries, ICDAS II index

## Abstract

Studies of the dental status of children with cerebral palsy (CP) indicate a high prevalence and intensity of damage to the hard tissues of the teeth. The risk of developing dental diseases is known to increase significantly as the severity of neurological symptoms increase. The purpose of the study was to assess the incidence of dental caries using the International Caries Detection and Assessment System (ICDAS II) criteria in children with organic diseases of the nervous system depending on the severity of motor impairment. A number of 122 children (mean age 8.8±3.7 years) with spastic forms of cerebral palsy were examined. They were divided into groups according to the Gross Motor Function Classification System – Expanded & Revised (GMFCS-ER). All patients underwent a neurological examination, and the state of dental caries was determined using the ICDAS II criteria. In children with cerebral palsy, lesions of the occlusal surfaces of the teeth predominate, lesions of the proximal surfaces appeared to be three times less, but more than three times higher than in healthy children. Higher intensity of the carious process and the frequency of deep cavities are observed in children with cerebral palsy with severe motor impairment, according to GMFCS-ER. Establishing the features of caries development in children with cerebral palsy depending on the severity of neurological symptoms according to the ICDAS II system is an essential factor in determining the direction of preventive measures that should be taken for this group of children.

## Introduction

Nowadays, infantile cerebral paralysis (ICP) or cerebral palsy (CP) remains one of the most common neurological pathologies, most often resulting in the disability of patients under 18 [1–3]. One of the major clinical symptoms of CP is motor function impairment associated with retarded development and improper formation of static-kinetic reflexes, tonus pathology, and paresis [4–9].

Examination of the dental status of children with CP is indicative of a wide occurrence and intensity of lesions of their hard dental and periodontal tissues. Some studies have demonstrated that the risk of development of dental diseases increases reliably with the increase of manifestation of neurological symptoms [10–15]. This can happen due to many factors, including motor and coordination impairments, limited possibility to take care of the oral cavity, resulting in insufficient individual hygiene [16, 17].

Considering the high occurrence of dental disorders, difficulty to implement traditional therapeutic and preventive measures, and the considerable effect of dental pathology on the quality of life of children with organic lesions of the nervous system, detection of caries lesions in this group of children depending on the intensity of motor impairment remains an important issue.

Therefore, the purpose of our study was to assess the incidence of dental caries using the International Caries Detection and Assessment System (ICDAS II) criteria in children with organic diseases of the nervous system depending on the severity of motor impairment.

## Material and Methods

A number of 122 children (mean age 8.8±3.7 years) with spastic types of cerebral palsy were examined at the Regional Center of Medical-Social Rehabilitation of Children with Organic Lesions of the Nervous System Chernivtsi, Ukraine. The children with CP were divided into groups according to the Gross Motor Function Classification System – Expanded & Revised (GMFCS-ER) [18]: the first group included 23 children (18.9%) performing gross motor skills without restrictions, the second group included 26 (21.3%) children performing gross motor skills with restrictions; the third group included 26 (21,3%) children walking by means of a hand-held mobility device; the fourth group included 25 (20.5%) children able to walk with physical assistance, and the fifth group included 22 (18.0%) children that were manual wheelchair users. The group of comparison consisted of 80 practically healthy children.

Caries dental lesions were assessed in all the children using the International Caries Detection and Assessment System (ICDAS II) [19]. ICDAS II includes three codes to evaluate carious changes in the enamel and three codes to evaluate changes in the dentin at an ever-increasing rate of their intensity (Table 1). An essential feature of the system is the possibility to assess primary changes in the available enamel.

**Table 1.** ICDAS II codes and their description in order to assess primary caries of the coronal part of the tooth [20].

**Code**	**Major criteria to find caries in the coronal surface of the tooth**
**0**	Intact surface
**1**	Primary visual changes of the enamel (visualized only after drying the enamel surface for 5 seconds) or changes of the enamel color in the fissures visual on the wet or dry surface of the tooth
**2**	Distinct visual changes in the enamel
**3**	Local enamel integrity damage without signs of dentin involvement into caries focus
**4**	Dark shadow of the afflicted dentin available
**5**	Visible carious cavity with bare dentin
**6**	Visible big carious cavity with bare dentin

According to the criteria, code 0 (ICDAS=0) corresponds to an intact tooth, 1 and 2 characterize initial lesions of the dental enamel (focal demineralization), and 3 corresponds to visible lesions of the dental enamel (superficial caries). Codes 4, 5 and 6 represent carious dental lesions. The criteria are developed considering the fact that every point corresponds to a specific degree of the lesion (intensity) of the hard dental tissues, and a direct correlation between points and intensity of lesion determined by pathomorphological methods is evidenced [19–21].

We assessed the absolute and relative number of teeth possessing different codes according to ICDAS II in different groups of children, as well as the absolute and relative amount of dental surfaces among all the surfaces examined, which enables to determine prevailing caries location among the group of children involved into the study, and, to some extent, make recommendations concerning therapeutic-preventive measures. The data obtained were statistically processed by means of the applied programs Microsoft Excel 2010®, Biostat®, Statistika® 7.0, and Student’s t-test (paired and unpaired).

## Results and Discussion

 The majority of the children examined were diagnosed with spastic forms of CP: 40 (32.8%) children had spastic diplegia, 25 (20.5%) had a hemiparetic form, 6 (4.9%) had spastic triparesis, 34 (27.9%) had spastic tetraparesis; hyperkinesis was diagnosed in 10 children (8.2%) and ataxic syndrome in 7 (5.7%) children.

Dental examination of children with organic nervous system lesions found that caries occurrence was 100%, contrary to healthy children from the comparison group where this index was 68.7%. An average value of caries intensity by the dt, DMF+dt, and DMF indices in children with CP was 6.27±1.19, which is 2.3 times higher than healthy children (2.72±1.17; p=0.038).

It should be noted that children with CP had a rather high percentage of extracted teeth: 1.11% (7 teeth) in children from the third group, 1.77% (11) in children from the 4^th^ group, 1.69% (9) in children from the fifth group. At the same time, teeth were not extracted in children from the control group or those with CP from the first and second groups. First of all, it indicates that teeth are decayed more intensively in children with pronounced mental and motor impairments; secondly, the lack of a proper level of (or access to get) oral cavity hygiene and retention of teeth in children is more intense in children with severe general conditions due to subjective and objective reasons.

At the same time, the number of filled teeth in children with CP and healthy ones did not differ reliably: 82 teeth (2.8%) among all the examined teeth in children with CP and 43 (2.3%) in somatically healthy children. Such an amount of treated teeth in children with CP is not indicative of proper oral hygiene since reliably higher indices of deep carious with their complications were found in children with CP with intense motor impairments, according to the data of our study.

**Figure 1.**
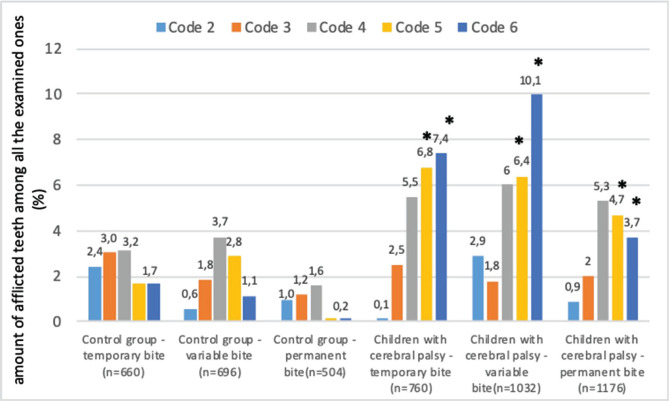
ICDAS II codes frequency in children with CP and practically healthy ones. n – number of examined teeth; * – reliable differences from the indices of practically healthy children.

Analysis of caries lesions according to the ICDAS II criteria in children with CP has demonstrated the following:

• Code 0 was registered in 73.2% of cases among all the examined teeth. In children from the control group, code 0 was registered in 87.97% of cases, which corresponds to the difference between caries occurrence in the patients with CP and without it;
• Code 1 was not practically registered in the groups of children with CP and practically healthy children. It might be associated with certain difficulties in examining sick children and finding inconsiderable damage of the normal condition of the dental enamel;
• Code 2 was found in 1.43±1.66% of all the examined teeth of children with CP compared to 1.32±0.72% in the control group (p>0.05);
• There were no reliable differences found in the percentage of teeth with codes 3 and 4 between children from the control group and those with CP: code 3 was found in 1.72±1.41% of children with CP compared to 2.02±0.66% in healthy ones;
• Code 4 was found in 4.49±1.54% in children with CP compared to 2.83±0.83% in the control group. Though in children with organic lesions of the nervous system, the percentage of teeth with code 4 was 3.2 times higher as compared to the similar index in healthy children, which is indicative of a tendency to a higher occurrence of hidden carious cavities and, thus, to progressing carious process in this group of patients [22].
• Regarding codes 5 and 6, we found reliable differences between sick and practically healthy children: code 5 was 3.8 times more frequent in children with CP compared to healthy ones – 5.91±1.84% vs. 1.58±0.92%; respectively (p<0.05). Code 6 was 7.5 times more frequent in children with CP compared to healthy children (7.52±3.17% vs. 1.03±0.54%, respectively; p<0.05). Such correlations remained valid during all occlusion periods (Figure 1), and the difference between indices in the main and control groups was found to be the most significant in children with transitional and temporal dentition.

**Figure 2.**
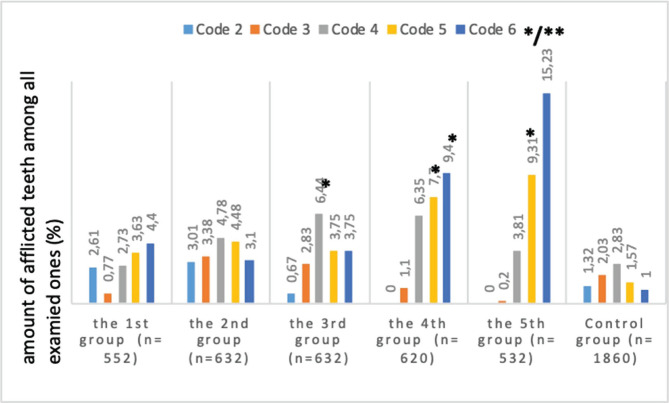
ICDAS II codes frequency in children with CP and according to the intensity of motor impairment. n – number of examined teeth; * – reliable differences from the indices of practically healthy children, ** – reliable differences from the indices of children with CP belonging to the first group.

Figure 2 shows comparative characteristics of carious lesions of the teeth according to the ICDAS II criteria in children with CP depending on the intensity of motor impairment.

Code 4 was found to be reliably higher (2.3 times as much) in children from the third group compared to the control group (6.44±1.81% vs. 2.83±0.83%, respectively; p=0.027). In children from the fourth and fifth groups who suffered from severe motor impairment, code 5 was found in a reliably higher percentage of children in comparison with healthy children (7.7±2.45% in patients from the fourth group; p=0.023 and 9.31±2.34% in patients from the fifth group vs. 1.57±0.92; p=0.0028 in the control group). The same was applicable for code 6 (9.36±4.10%; p=0.048 in the fourth group, 15.23±5.1%; p=0.0077 in the fifth group and 1.03±0.54% in the control group), which is indicative of a considerable occurrence of extensive caries of the dentin in these groups of patients. Differences between high codes in children from the main and control groups are registered mostly at the expense of those children who suffer from severe motor impairment.

Analysis of differences among children from different groups by the intensity of motor impairment found a reliable difference between code 6 in the fifth group of patients with CP and the first group with inconsiderable motor impairment: code 6 was 4.2 times more frequent in patients from the fifth group in comparison with patients from the first group (15.23±5.1% vs. 3.62±1.59%, respectively; p=0.034). It can be indicative of increased intensity of an active carious process with the formation of extensive carious cavities closely correlated with motor impairment in children with CP.

**Figure 3.**
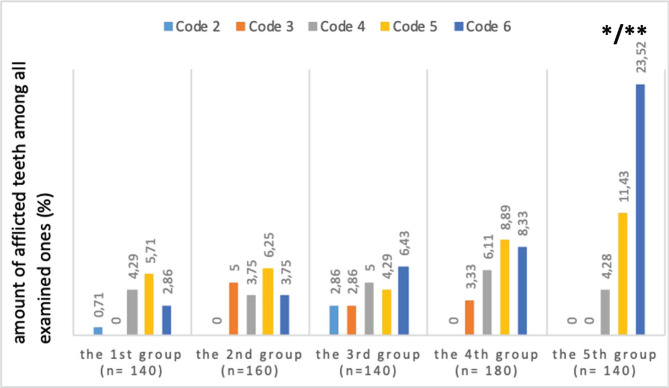
Occurrence of ICDAS II codes in children with CP from different groups according to the intensity of motor impairment in temporary dentition. n – number of examined teeth; * – reliable differences from the indices of children with CP from the first group; ** – reliable differences from the indices of children with CP from the second group.

**Figure 4.**
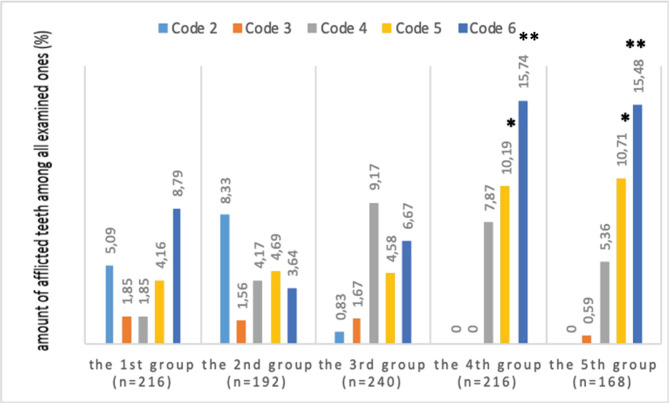
Occurrence of ICDAS II codes in children with CP from different groups according to the intensity of motor impairment in transitional dentition. n – number of examined teeth; * – reliable differences from the indices of children with CP from the first group; ** – reliable differences from the indices of children with CP from the second group.

**Figure 5.**
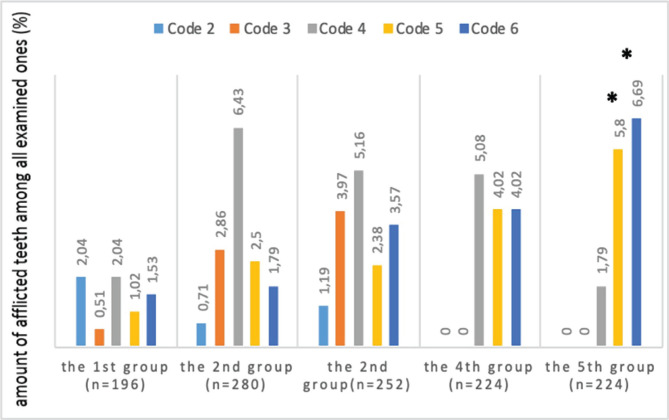
Occurrence of ICDAS II codes in children with CP from different groups according to intensity of motor impairment in permanent dentition. n – number of examined teeth; * – reliable differences from the indices of the first group.

Similar tendencies were observed in children with temporary, transitional, and permanent dentition (Figures 3–5). An extremely high occurrence of code 6 (deep, extensive carious cavities) in children from the fifth group with temporary dentition and low indices of enamel caries is of special attention. It is indicative of a rapid progression of carious cavities in this group of children (Figure 3).

Dentine caries prevails considerably in children with transitional dentition (Figure 4). Superficial caries or enamel caries were found only among the examined children from groups 1, 2 and 3. It happened mainly at the expense of initial caries of permanent teeth that erupted recently. Its absence in children with more intensive motor impairments is indicative of a rapid progression of carious lesions with the formation of dentine caries.

In permanent dentition, the teeth with code 4 prevailed. Though, with the increase of the degree of motor impairment functions (groups 3, 4 and 5), code 4 was gradually substituted by codes 5 and 6 (visible dentine caries), which was indicative of increased severity of caries in children with deterioration of their motor activity (Figure 5).

Analysis of caries structure on the surface of non-filled teeth in children with CP (Table 2) determined that the grinding surface of teeth was afflicted most frequently (15.17%) (code 2 and higher). It is stipulated by the anatomical shape, depth, and width of the grinding teeth fissures, low level of mineralization in comparison with other parts of the enamel, and poor moistening with saliva [23]. At the same time, the highest caries indices of the grinding surfaces of the teeth were found in patients from the fourth (125 children, 23.15%) and fifth (135 children, 23.3%) groups. It can be indicative of the inability of children from these groups to clean their teeth carefully, even on accessible surfaces.

**Table 2.** Caries affliction of the teeth surfaces by ICDAS II in children with cerebral palsy from different groups according to the Scale of major motor functions in comparison with healthy children.

**ICDAS II codes**	**Surfaces of the tooth**	**Groups of children with CP according to the Scale of major motor functions**					**All the children with CP** **n (%)**	**Practically healthy children n (%)**
		**1; n(%)**	**2; n (%)**	**3; n (%)**	**4; n (%)**	**5; n (%)**		
**2**	**A**	0	1 (0.08)	0	0	0	1 (0.02)	0
	**G**	7 (1.26)	17 (2.69)	6 (0.95)	0	0	30 (1.01)	19 (1.02)
	**O**	0	0	0	0	0	0	0
	**V**	0	0	0	0	0	0	0
**3**	**A**	1 (0.09)	1 (0.08)	9 (0.71)	0	0	11 (0.19)	10 (0.27)
	**G**	3 (0.54)	7 (1.11)	5 (0.79)	6 (0.96)	1 (0.19)	22 (0.74)	28 (1.5)
	**O**	0	0	0	0	0	0	0
	**V**	0	10 (1.58)	4 (0.63)	0	0	14 (0.47)	3 (0.16)
**4**	**A**	10 (0.91)	37 (2.93)	30 (2.37)	18 (1.45)	11 (1.03)	106 (1.79)	11 (0.3)
	**G**	4 (0.72)	7 (1.11)	24 (3.79)	17 (2.74)	8 (1.5)	60 (2.02)	36 (1.94)
	**O**	0	4 (0.63)	4 (0.63)	0	0	8 (0.26)	0
	**V**	0	4 (0.63)	4 (0.63)	0	0	8 (0.26)	0
**5**	**A**	5 (0.45)	25 (1.98)	8 (0.63)	20 (1.61)	14 (1.31)	72 (1.21)	14 (0.38)
	**G**	10 (1.81)	25 (3.95)	21 (3.32)	47 (7.58)	43 (8.08)	146 (4.92)	32 (1.72)
	**O**	0	12 (1.89)	3 (0.47)	10 (1.61)	5 (0.94)	30 (1.01)	5 (0.27)
	**V**	4 (0.72)	12 (1.89)	3 (0.47)	10 (1.61)	5 (0.94)	34 (1.15)	5 (0.27)
**6**	**A**	16 (1.44)	0	1 (0.08)	38 (3.06)	48 (4.51)	103 (1.73)	10 (0.27)
	**G**	26 (4.71)	15 (2.37)	24 (3.79)	55 (8.87)	72 (13.53)	192 (6.48)	21 (1.13)
	**O**	8 (1.31)	0	0	17 (2.74)	23 (4.32)	48 (1.62)	5 (0.27)
	**V**	8 (1.31)	0	0	17 (2.74)	23 (4.32)	48 (1.62)	5 (0.27)

Occurrence of codes is indicated different from 0; n – number of surfaces of the teeth having the indicated code; **A** – approximal surface; **G** – grinding surface; **O** – oral surface; **V** – vestibular surface.

In children with CP, approximal caries were found in 4.94% of the general amount of the examined surfaces of the teeth. Analysis of caries on the approximal surfaces found that the highest percentage of this index was found among children with CP and intensive motor impairment: 6.12% in the fourth group and 6.85% in the fifth group. Approximal surfaces of the molars were afflicted most often, which is explained by the practically complete absence of interdental hygiene of the examined children. Code 4 was most often registered on the approximal surfaces (hidden caries), codes 5 and 6 on the grinding surfaces (often spread from the approximal cavities to the grinding surface with advanced caries process). Despite this, they were not less frequent than the occurrence of approximal caries. High occurrence of caries on the approximal surfaces is known to be associated with peculiarities of the anatomical structure, accumulation of food debris and unsatisfactory oral hygiene, formation of aggressive dental plaque in the natural depressions of teeth, intake of refined carbohydrates, influence of negative factors of the underlying disease, and longer periods of hypomineralization in comparison with the smooth surfaces of the teeth [24–30].

In some cases, the carious lesion was associated with the formation of defects on the vestibular and oral surfaces of the teeth in children with CP, which was 5 times higher than that of occurrence of those defects in practically healthy children – 104 children (3.5%) vs. 13 (0.7%) among all the examined teeth, respectively. Depth of vestibular defects was different: in some cases, occlusive and vestibular cavities remained isolated one from another; in some of them, rapid destruction of hillocks, consolidation of cavities, and enamel loss on the greater part of the occlusive and smooth surfaces occurred, followed by further destruction of the dentin, development of caries complications with complete loss of the crown, especially in children with severe motor impairments.

Thus, lesions of the occlusive surfaces of the teeth prevail in children with CP (practically twice as many as those in healthy children); yet, lesions of the approximal surfaces were three times less. However, this index was increased by three times compared to healthy children. The high occurrence of occlusive caries in temporary dentition can be explained by quick destruction of the grinding surface of the molars at the expense of its hypomineralization during intrauterine development of a child caused by the factors stipulating the formation of neurological pathology. Prevailing of occlusive caries over approximal ones in the transitional and permanent dentition can be explained by the lack of close space between teeth at the expense of incomplete formation of dentition. Smooth surfaces of the teeth are afflicted in the areas of physiological hypomineralization, and in the area of the neonatal line in particular, in case proper oral hygiene is lacking.

Finding peculiarities of caries development in children with CP depending on the severity of neurological symptoms is an important factor in order to determine the direction of preventive measures for this group of children.

## Conclusions

We found a higher frequency of caries process among children with CP and severe motor impairment. Surfaces of the teeth with deep caries (codes 5 and 6 according to ICDAS II) are more often found among children with CP and severe motor impairments. Occlusive caries prevails among both children with CP and somatically healthy ones, though lesions are found on all the surfaces of the teeth.

## Acknowledgments

### Ethical approval

The approval for this study was obtained from the Ethics Committee of the HSEEU Bukovinian State Medical University, Ukraine (approval ID: 11-08.11.2019),

### Consent to participate

Written informed consent was obtained from the patient’s parents.

### Conflict of interest

The authors declare that there is no conflict of interest.
